# Radix Rehmanniae Praeparata (Shu Dihuang) exerts neuroprotective effects on ICV-STZ-induced Alzheimer’s disease mice through modulation of INSR/IRS-1/AKT/GSK-3β signaling pathway and intestinal microbiota

**DOI:** 10.3389/fphar.2023.1115387

**Published:** 2023-02-08

**Authors:** Yunfang Su, Ningning Liu, Ruiqin Sun, Jinlian Ma, Zhonghua Li, Pan Wang, Huifen Ma, Yiran Sun, Junying Song, Zhenqiang Zhang

**Affiliations:** ^1^ Henan Engineering Research Center for Prevention and Treatment of Major Chronic Diseases with Chinese Medicine, Academy of Chinese Medical Sciences, Henan University of Chinese Medicine, Zhengzhou, China; ^2^ The First Affiliated Hospital of Henan University of Chinese Medicine, Zhengzhou, China

**Keywords:** Radix Rehmanniae Praeparata, Alzheimer’s disease, ICV-STZ, insulin signaling pathway, intestinal microbiota

## Abstract

Radix Rehmanniae Praeparata (RRP, Shu Dihuang in Cinese) is widely used as primal medicine in Chinese herbal formula for the treatment of Alzheimer’s disease (AD). However, the underlying mechanism of RRP for AD remains unclear. The aim of this study was to investigate the therapeutic effect of RRP on intracerebroventricular injection of streptozotocin (ICV-STZ)-induced AD model mice and its potential mechanism. ICV-STZ mice were continuously gavaged with RRP for 21 days. The pharmacological effects of RRP were evaluated by behavioral tests, brain tissue H&E staining and hippocampal tau protein phosphorylation levels. The expression levels of insulin receptor (INSR), IRS-1, pSer473-AKT/AKT and pSer9-GSK-3β/GSK-3β proteins in hippocampal and cortical tissues were detected by Western-blot method. The 16S rRNA gene sequencing was used to analyze the changes of intestinal microbiota in mice. The compounds in RRP were analyzed by mass spectrometry and their binding ability to INSR proteins was detected by molecular docking. The results showed that RRP ameliorated cognitive dysfunction and neuronal pathological changes of brain tissue in ICV-STZ mice, reduced tau protein hyperphosphorylation, INSR, IRS-1, pSer473-AKT/AKT, and pSer9-GSK-3β/GSK-3β levels in hippocampal and cortical tissues. Meanwhile, RRP reversed ICV-STZ-induced dysregulation of intestinal microbiota in AD mice. Mass spectrometry analysis showed that the RRP consisted mainly of seven compounds, namely Acteoside (Verbascoside), 5-Hydroxymethyl-2-furaldehyde (5-HMF), Apigenin7-O-glucuronide, Icariin, Gallic acid, Quercetin-3β-D-glucoside, and Geniposide. Molecular docking results further indicated that the compounds in RRP have binding ability to INSR protein and potential multiple synergistic effects. RRP ameliorates cognitive dysfunction and brain histopathological changes in AD mice. The mechanism of RRP ameliorating AD may be related to the regulation of INSR/IRS-1/AKT/GSK-3β signaling pathway and intestinal microbiota. This study supports the potential anti-AD efficacy of RRP and initially reveals the pharmacological mechanism of RRP, providing a theoretical basis for further clinical application of RRP.

## 1 Introduction

Alzheimer’s disease (AD), the most common neurodegenerative disease, is one of the greatest medical challenges of this century and is becoming increasingly prevalent worldwide (Hickman et al., 2016). The number of AD patients worldwide exceeded 50 million in 2018 (Hodson, 2018). In 2050, the number of people with AD will reach 131 million worldwide and the socioeconomic cost will reach $9.12 trillion (Jia et al., 2018). According to the World Alzheimer Report of 2015, the world annual growth rate of dementia patients was 1.033 and the annual growth rate of the consumer price index was 1.031. (Prince et al., 2015). So, the patient number of AD probably is 60 million and the socioeconomic cost is $1.4 trillion in this year (2023). The main clinical manifestations of AD include progressive memory loss, impaired judgment, disorientation, behavioral abnormalities, and social dysfunction. The pathogenesis of AD is complex, with typical pathological features including extracellular senile plaques (SP) formed by intracerebral β-amyloid (Aβ), intracellular neurogenic fiber tangles (NFTs) caused by tau protein hyperphosphorylation, and progressive brain neuronal loss (Gong et al., 2018). There are limited drugs available clinically to treat AD, which only relieve symptoms but cannot stop disease progression (Cummings et al., 2020). Common clinical anti-AD drugs include the cholinesterase inhibitors donepezil, carboplatin and galantamine, and the glutamate receptor antagonist memantine. New anti-AD drugs marketed in recent years include GV-971, an intestinal flora modulator, and Aducanumab, an Aβ inhibitor. Aducanumab is currently very controversial in clinical application (Yeo-Teh and Tang, 2023).

AD is also known as “type 3 diabetes” (de la Monte et al., 2018). The focus on glucose metabolism is considered a new trend in AD research (Kuehn, 2020). The insulin signaling pathway plays an important role in the onset and development of AD and is expected to be a beneficial target for the treatment of AD (Kellar and Craft, 2020). The insulin signaling pathway mainly consists of the insulin receptor (INSR), insulin receptor substrate 1 (IRS-1) and the downstream of the phosphoinositide 3-kinase (PI3K) signaling pathway. Insulin or insulin growth factor-1 (IGF-1) regulate the phosphorylation of glycogen synthase kinase-3β (GSK-3β) and inhibit the production of tau hyperphosphorylation and the accumulation of Aβ by altering their activity through activation of INSR, IRS-1 and PI3K/AKT (Verdile et al., 2015). So, INSR/IRS-1/AKT/GSK-3β signaling pathway plays an important role in the process of AD. Studies have shown that modulation of neuronal insulin signaling pathways can effectively improve AD (Xiong et al., 2020; Tyagi and Pugazhenthi, 2021). A growing number of studies have shown that dysbiosis of the gut flora is prevalent in AD patients, with changes in phylum levels concentrated in the phylum of Bacteroidetes, Firmicutes, and Actinobacteria (van Olst et al., 2021). The mechanisms by which intestinal flora contribute to AD are also closely related to inflammation, with bacterial metabolites and toxins affecting the central immune system through regulation of peripheral and central immune cells, cytokine secretion, and blood-brain barrier (BBB) function (van Olst et al., 2021). Among the studies on intestinal microbiota-based treatment of AD, various therapeutic tools can exert anti-AD effects by modulating intestinal microbiota, such as GV-971 (Wang et al., 2019), the Chinese herbal formula Huanglian Jiexue Tang (Gu et al., 2021) and *Clostridium butyricum* (Sun et al., 2020).

The Chinese medicine Rehmannia (Sheng Dihuang in Chinese) is the dried root of the perennial herb Rehmannia glutinosa Libosch of the scrophulaceae family. Radix Rehmanniae Praeparata (RRP, Shu Dihuang in Chinese) is obtained by steaming Rehmannia with wine to modify its medicinal properties (Meng et al., 2017). RRP is known for its “*yin*” nourishing effects such as lowering blood sugar (Xiang et al., 2021) and enhancing memory (Cui et al., 2002). According to Chinese medicine, the core pathogenesis of AD is “*kidney yin deficiency*”. RRP has a therapeutic effect on type II diabetes mellitus (T2DM) and can improve peripheral insulin resistance (Xie et al., 2011). Also, RRP has been reported to improve intestinal microbiota in obese patients (Han et al., 2015). Catalpol, a component of RRP, has neuroprotective effects on AD mice (Huang et al., 2016). Our previous study showed that Liuwei Dihuang pill (a Chinese herbal formula containing RRP) could improve cognitive functions through inhibiting neuroinflammation in AD mice (Song et al., 2022). However, it is not clear whether RRP can improve brain insulin signaling in AD and the underlying mechanisms. Intracerebroventricular injection of streptozotocin (ICV-STZ) induced mice is commonly used in a sporadic AD model that exhibits impaired insulin signaling in the brain (Grieb, 2016). The aim of this study was to investigate whether RRP ameliorates ICV-STZ-induced AD pathology and to explore the underlying mechanisms.

In this study, we explored the efficacy of RRP using ICV-STZ-induced mice and examined the expression levels of INSR/IRS-1/AKT/GSK-3β signaling pathway related proteins in brain tissue. Meanwhile, the intestinal microbiota of mice in each group was analyzed. In addition, we analyzed the compounds in RRP and assessed the interaction of compounds with INSR proteins by molecular docking. The results showed that RRP has anti-AD effect and the mechanism maybe related to the regulation of INSR/IRS-1/AKT/GSK-3β signaling pathway and intestinal microbiota.

## 2 Materials and methods

### 2.1 Materials

RRP extracted powder (XP-20201022, tested and approved) was purchased from Shaanxi Sypal Bio-technique Co., LTD. The main materials involved in this study included STZ (S0130, SIGMA), isoflurane (R510-22, Shenzhen Ruiwade Life Technology Co., LTD), Hematoxylin-Eosin Staining Kit (G1120, Solarbio), DAPI (Roche Life Science, Indianapolis), primary antibodies β-Actin (42 kDa, BM0627, and BOSTER), INSR (95 kDa, GTX101136, and GeneTex), p^Ser404^-tau, IRS-1, AKT, p^Ser473^-AKT, GSK-3β, and p^Ser9^-GSK-3β (48–63 kDa, 20194T; 180 kDa, 2382S; 60 kDa, 9272S; 60 kDa, 4060S; 46 kDa, 12456T; 46 kDa, 5558T, Cell Signaling Technology), Horseradish peroxidase (HRP)-linked secondary antibodies against rabbit and mouse IgG of the primary antibodies (ZB-2301; ZB-2305, Beijing Zhong Shan-Golden Bridge Biological Technology) and Goat anti-Rabbit IgG (H + L) Cross-Adsorbed Secondary Antibody, Alexa Fluor™ 594 (A-11012, Thermo Fisher Scientific).

### 2.2 Animals and drug treatment

**FIGURE 1:**
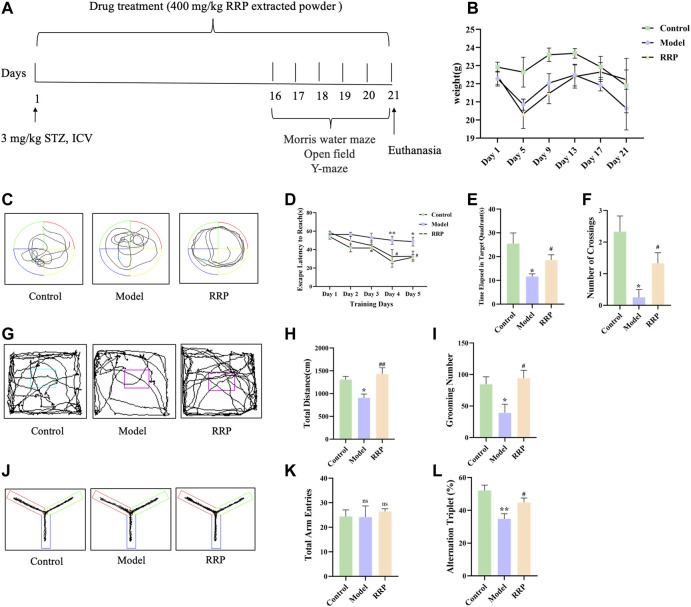
Experimental design, the weight changes and behavior experiments of mice. **(A)** 3 mg kg^−1^ STZ was injected into the lateral ventricle to induce AD model mice. Mice of control group were injected with an equal volume of sodium citrate buffer. Mice in RRP-treated group were continuously gavaged with 400 mg/kg RRP for 21 days. The control and model groups were gavaged with equal volumes of PBS. Behavior tests were performed on the last 5 days. Tissues were collected after euthanasia. **(B)**. The weight changes of mice during the experiment. **(C)**. Morris water maze swimming trajectories of mice in each group; **(D)**. Morris water maze evasion latency; **(E)**. Time elapsed in target quadrant in the Morris water maze; **(F)**. The number of platform crossings in Morris water maze; **(G)**. Open field activity trajectories of mice in each group; **(H)**. Total distance; **(I)**. Grooming times; **(J)**. Y maze activity trajectory of mice in each group; **(K)**. The total number of Y maze arm entries in each group; **(L)** Spontaneous altercountry accuracy of Y maze. * model group compared with the control group, # RRP group compared with the model group, same as in Figures 3–5.

Six-to eight-week-old male C57BL/6N mice (weight 20–30 g) were purchased from Weitong Lihua Laboratory Animal Technology Co., LTD. (Beijing, China). The experimental animal license number is SCXK (jing) 2021-0006. The animal room was maintained at room temperature of (22 ± 2)°C, humidity of 60%, 12:12 h light/dark cycle conditions, free diet and water. The animal experimental design was in accordance with the requirements of the Experimental Animal Ethics Committee of Henan University of Chinese Medicine (DWLLGZR202202147). After 1 week of adaptive feeding, AD models were prepared by lateral ventricular injection of 3 mg kg^−1^ STZ, which is an optimized animal model in AD studies resulting in hyperphosphorylation of tau (Yang et al., 2020; El Sayed et al., 2021; Zhao et al., 2021). STZ (30 μg μl^-1^) was dissolved in sodium citrate (pH 4.2, 1% w/v) prepared before injection. For sodium citrate buffer preparation, 2.1 g citric acid (FW: 210.14) was added to 100 mL of distilled water to form liquid A, and 2.94 g sodium citrate (FW: 294.10) was added to 100 mL of distilled water to form liquid B. When used, liquid A and liquid B were mixed 1:1 to form sodium citrate buffer. The mice were fixed in a brain stereotaxy instrument. After anaesthetized by anesthesia machine, the anterior fonthalle was exposed through a median incision at the top of the lateral ventricle. The right lateral ventricle was slowly injected with STZ for 5 min and left it for another 5 min before slowly withdrawing the needle. All procedures were performed in a sterile environment. The wounds were sutured after antiseptic treatment with penicillin. The sham-control group (control group) was injected with an equal volume of sodium citrate buffer. Control group, model group and RRP-treated group were set up with 10 mice in each group. RRP extracted powder was dissolved in saline and mice were administered intragastrically with a dose of 400 mg kg^−1^ d^−1^ for 21 days according to the suggestion of Pharmacopoeia of the People’s Republic of China and the dose optimization was performed in the previous study (Li et al., 2021). Mice in control and model groups were given an equal volume of saline. The experimental design of this study is shown in Figure 1A. The weight of the experimental animals was measured during the experiment every 4 days.

### 2.3 Behavior experiments

Behavioral experiments were performed for the last 5 days of the experiment. In the Morris water maze experiment (Song et al., 2022), mice were placed in water facing the wall of the pool from four different entry points and the time taken to climb onto the platform (i.e., escape latency) was recorded for five consecutive days. On day 6, the platform was removed and the mice were placed in the water at the same entry point. The residence time in the target quadrant and the number of times the mice crossed the platform within 60 s were recorded. In the open field experiment (Zhang et al., 2022), the mice moved freely for 5 min, and the total movement distance and the number of grooming were recorded. In the Y-maze experiment (Kraeuter et al., 2019), the mice were placed at the intersection of three maze arms in turn and moved freely for 5 min. The total number and order of mice entering the maze arms were recorded, and the accuracy of spontaneous alternation was calculated.

### 2.4 Histological examination

After behavioral testing, mice were anesthetized with isoflurane and brain tissues were collected in 4% paraformaldehyde. Hematoxylin-eosin (H&E) staining was performed to compare the histopathological changes in each group. All brain tissues were paraffin-embedded and sectioned according to the conventional method (El Sayed et al., 2021). 5 μm thick tissue sections were prepared for H&E staining. A microscope (Axioscope 5, ZEISS, Germany) was used to observe and take tissue images. The histopathological changes in the brain of each group of mice were observed.

### 2.5 Immunofluorescence assay

Paraffin sections in 2.4 were dewaxed with xylene, rehydrated, treated with 0.5%–1% protease, and 5% skim milk closed with 1.5 h p^Ser404^-tau and Alexa Fluor 594 goat anti-mouse IgG (H + L) were used as primary and secondary antibodies, respectively, and finally nuclear staining was performed with DAPI (Ma et al., 2022). A microscope (Axioscope 5, ZEISS, Germany) was used to observe and take images of the tissues.

### 2.6 Western-blot assay

The hippocampal and cortical tissues of each group of mice were added with appropriate amount of tissue lysis solution, ground in a grinder at 4°C, lysed on ice for 30 min, and centrifuged at 4°C for 15 min at 12000 r min^−1^. The total proteins were collected, and protein concentrations were detected by BCA kit. Western-blot assay was performed to detect p^Ser404^-tau, INSR, IRS-1, p^Ser473^-AKT/AKT, p^Ser9^- GSK-3β/GSK-3β levels. β-Actin was used as an internal control for data analysis (Song et al., 2022). The primary antibody was diluted with 5% skim milk powder (1:1000 diluted) for 1 h to better bind non-specific proteins. The primary antibody was incubated overnight with 5% BSA solution and washed with 1✕TBST buffer solution 3 times for 10 min each to avoid the influence of phosphoric acid groups. The secondary antibody was diluted with 1✕TBST (1:8000) and incubated slowly on a shaker at room temperature for 1 h. After 3 times of washing, the protein bands were observed and photographed.

### 2.7 16S rRNA gene sequencing of intestinal microbiota in cecal contents

The contents of the cecum were collected from five mice per group after anesthesia. All samples were immediately placed in sterile tubes and stored at −80°C. Bacterial DNA was routinely extracted, primed to amplify a 16S rRNA gene V3-V4 variable region sequencing library, and sequenced on the Novaseq-PE250 (Illumina, Inc.) platform. The amplicon sequence variants (ASVs)/operational taxonomic units (OUT) were clustered using the DADA2 method combined with Vsearch method quality control to obtain the amplicon sequence variants (ASVs)/OUT abundance table with 100% similarity. Alpha diversity analysis (including chao1 index, Simpson index, shannon index, *etc.*) and species analysis (including species composition analysis and heat map analysis, *etc.*) were used to evaluate the abundance and diversity of the microbiota. Beta diversity analysis based on weighted UniFrac was used to evaluate the differences in microbiotic structure between samples (including principal coordinate analysis, PCOA analysis; non-metric multidimensional scaling, NMDS, *etc.*). Genus-level species composition heat maps were drawn for species clustering, in which samples were clustered according to the Euclidean distance of species composition data in UPGMA and ranked according to the clustering results. Statistical analysis of phylum and class level abundance among different groups was performed based on ASVs/OTU sequence abundance. 16S rRNA gene sequencing was performed by Suzhou PANOMIX Biomedical Tech Co., LTD. (Chen et al., 2022).

### 2.8 Analysis of compounds in RRP

200 mg of RRP extracted powder was added to 1 mL of 80% methanol solution and vortexed for 10 min. The sample were centrifuged at 4°C for 10 min at 20000✕g, and the supernatant was filtered and analyzed for composition. Mass spectrometry analysis was performed using a Q Exactive high-resolution mass spectrometer (Song et al., 2022). The global parameters were optimized, ion source: electrospray ionization (ESI); scan mode: Positive and negative ion switching scan; detection mode: Total mass/dd-MS2; resolution: 70000 (total mass), 17500 (dd-MS2); scan range: 100–1500 m/z; standby voltage: 3.8 kV (positive); capillary temperature: 300°C; data acquisition time: 30 min; chromatographic conditions: the column of AQ-C18, 150 × 2.1 mm, 1.8 μm, Welch. The mobile phase was water containing 0.1% formic acid (A) and methanol (B) at a flow rate of 0.30 mL/min. A gradient scheme was used: 98% A and 2% B for 0–1 min, 80% A and 20% B for 1–5 min, 50% A and 50% B for 5–10 min, 20% A and 80% B for 10–15 min, 5% A and 95% B for 15–27 min, 98% A and 2% B for 27–30 min. The injection volume was 3 μl The collected data were initially collated by CD2.1 (Thermo Fisher), and then searched and compared in a database (mzCloud).

### 2.9 Docking analysis of compounds in RRP with INSR protein

Docking the INSR protein with the compounds obtained in Section 2.8 using Discovery Studio software, detecting hydrogen bond formation, binding energy and binding sites, and evaluating the binding ability of the compound to the INSR.

### 2.10 Statistical analysis

Statistical analysis was performed using GraphPad Prism nine software. One-way ANOVA was performed on the experimental data. Data were expressed as M ± SD, and *p* < .05 was considered significant.

## 3 Results

### 3.1 RRP improves learning memory and spatial exploration in AD mice

The weight of the experimental animals was shown in the revised Figure 1B with no significant difference among groups. Figure 1C showed the Morris water maze swimming trajectories of mice in each group. The results of Morris water maze positioning navigation experiment showed that the escape latency of mice in each group gradually decreased with increasing training time and number. Compared with the control group, the evasion latency of the model group was significantly longer from day 4 (*p* < .01), and the evasion latency of the RRP-treated group was significantly shorter from day 4 compared with the model group (*p* < .05) (Figure 1D). The results of the spatial exploration experiment showed that elapsed time in targeted quadrant and the number of platform crossing was significantly reduced in the model group compared with the control group (*p* < .05, *p* < .05) (Figures 1E, F), and elapsed time in targeted quadrant and the number of platform crossing was significantly increased in the RRP-treated group compared with the model group (*p* < .05, *p* < .05) (Figures 1E, F). Open field activity trajectories of mice in each group were shown in Figure 1G. The results of open field test showed that the total distance of exercise was significantly lower in the model group than that in the control group (*p* < .05), and significantly increased in the RRP-treated group (*p* < .01) (Figure 1H). The number of grooming was significantly reduced in the model group compared to the control group (*p* < .05), while the number of grooming was significantly increased in RRP-treated group compared to the model group (*p* < .05) (Figure 1I). Figure 1J was Y maze activity trajectory of mice in each group. Y-maze test showed no difference in the total number of upper limb approaches in each group (Figure 1K). Spontaneous alternation accuracy was significantly lower in the model group compared to the control group (*p* < .01), and significantly higher in the RRP-treated group compared to the model group (*p* < .05) (Figure 1L).

### 3.2 RRP improves brain pathology in AD mice

**FIGURE 2:**
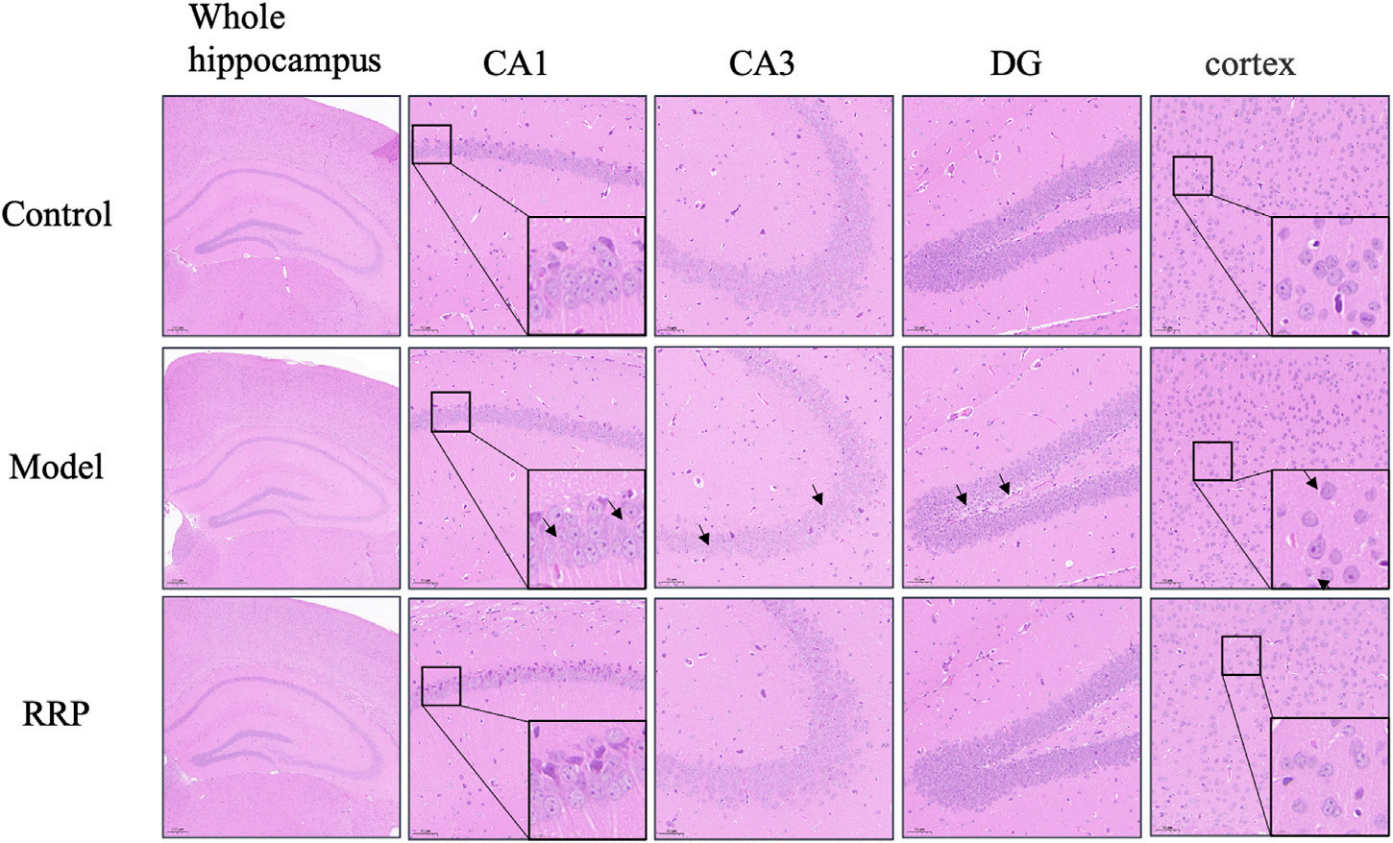
H&E staining of brain tissues of mice in each group. Neurons in control group of mice were normal structurally intact, well arranged and with clear nuclear staining. Neuronal cells were loosely arranged with karyopyknosis (as shown by the black arrow) in model group. RRP-treated group showed neatly arranged, evenly distributed and clearly structured. **(**The whole hippocampus is Magnification ×40; CA1, CA3, cortex is Magnification ×200, partial enlarged in CA1 and cortex is Magnification ×1400).

As shown in Figure 2, the hippocampal CA1, CA3, dentate gyrus (DG) and cortical neurons in the control group were structurally intact, well arranged and with clear nuclear staining, whereas neuronal cells in the model group were loosely arranged with karyopyknosis (as shown by the black arrow). RRP-treated group showed significant improvement in pathological changes in the hippocampus and cortex, with neuronal cells neatly arranged, evenly distributed and clearly structured.

### 3.3 RRP inhibits hippocampal tau protein phosphorylation in AD mice

**FIGURE 3:**
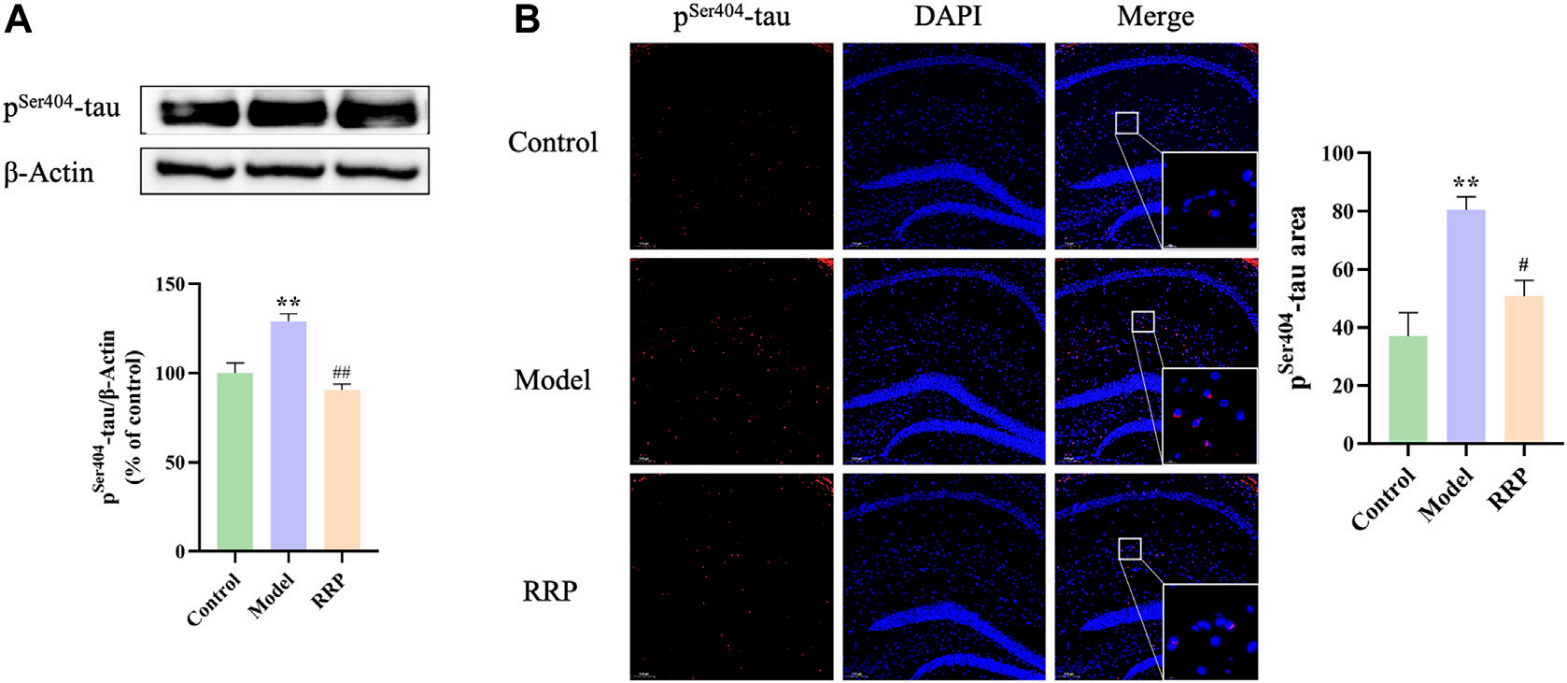
Western-blot and IF staining of p^Ser404^-tau protein in hippocampus of mice in each group. **(A)**. Expression levels of p^Ser404^-tau protein in hippocampus tissues; **(B)**. p^Ser404^-tau protein staining (red) and nuclei re-stained (blue) (Magnification ×100, partial enlarged is Magnification ×1000). Each bar with vertical line represents the mean ± SD of three mice per group.

Western-blot results showed that p^Ser404^-tau protein expression levels were significantly elevated in the model group compared with the control group (*p* < .01), and p^Ser404^-tau protein levels were significantly lower in the RRP-treated group compared with the model group (*p* < .01) (Figure 3A). Similarly, as shown by IF staining in Figure 3B, more p^Ser404^-tau protein was present in the brain tissue of model mice, which was significantly elevated compared to the control group (*p* < .01), while a small amount of p^Ser404^-tau protein was present in the brain tissue of RRP-treated mice, which was significantly lower compared to the model group (*p* < .05) (red, indicating p^Ser404^-tau protein positivity).

### 3.4 RRP inhibits INSR, IRS-1, p^Ser473^-AKT/AKT, p^Ser9^-GSK-3β/GSK-3β protein expression levels in the hippocampus, and cortex in AD mice

**FIGURE 4:**
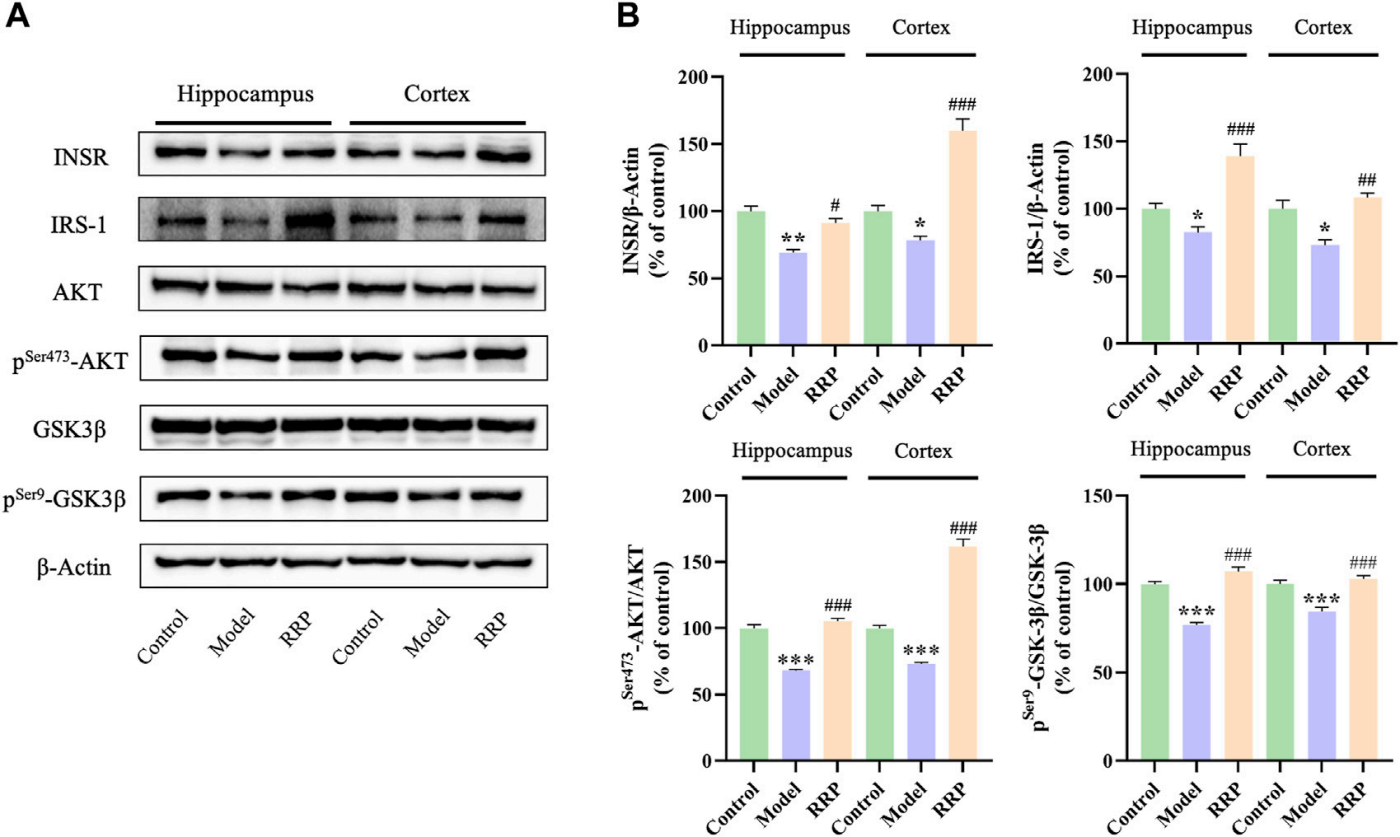
Western-blot of INSR, IRS-1, p^Ser473^-AKT/AKT, and p^Ser9^-GSK-3β/GSK-3β in hippocampus and cortex tissues of each group of mice. **(A)** Electrophoresis of INSR, IRS-1, AKT, p^Ser473^-AKT, GSK-3β, p^Ser9^-GSK-3β in hippocampus and cortex tissues of each group of mice. **(B)** Protein expression levels of INSR, IRS-1, p^Ser473^-AKT/AKT, and p^Ser9^-GSK-3β/GSK-3β in hippocampus of mice in each group. Each bar with vertical line represents the mean ± SD of three mice per group.

As shown in Figure 4, INSR, IRS-1 protein expressions were significantly decreased in hippocampal (*p* < .01, *p* < .05) and cortical (*p* < .05, *p* < .05) of mice in the model group compared to the control group, while the expression levels of INSR, IRS-1 proteins were significantly increased in hippocampal (*p* < .05, *p* < .001) and cortical (*p* < .001, *p* < .01) of mice in the RRP-treated group compared to the model group. Similarly, expression ratios of p^Ser473^-AKT/AKT, and p^Ser9^-GSK-3β/GSK-3β were significantly decreased in hippocampal (*p* < .001, *p* < .001) and cortical (*p* < .001, *p* < .001) of mice in the model group compared to the control group, while the ratios were significantly increased in hippocampal (*p* < .001, *p* < .001) and cortical (*p* < .001, *p* < .001) of mice in the RRP-treated group.

### 3.5 RRP reverses the intestinal microbiota changes of AD mice

#### 3.5.1 Analysis of alpha and beta diversity

**FIGURE 5:**
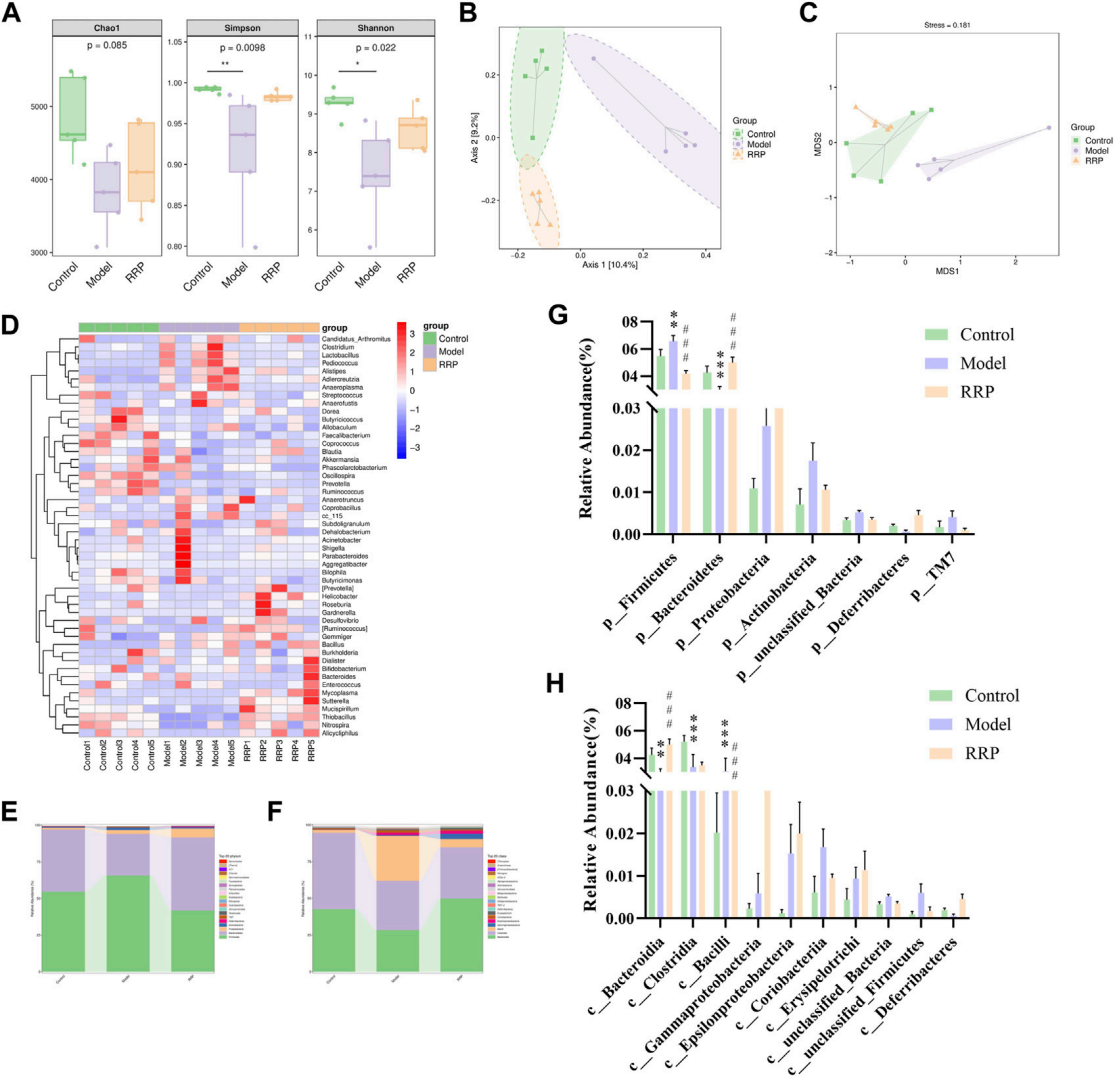
Analysis of intestinal microbiota in cecal contents of mice in each group. **(A)**. Chao1, Simpson and Shannon values of alpha diversity index in each group. **(B,C)**. PCOA and NMDS analysis of beta diversity; **(D)**. Genus-level species composition heatmap for species clustering. The red color blocks represent the abundance of the genus that is higher in this sample than in other samples, and the blue color blocks represent the abundance of the genus, that is, lower in this sample than in other samples; **(E–H)**. Bar graph of species composition at the phylum, class levels.

As shown in Figure 5A, alpha diversity showed that Chao1 values were decreased in the model group compared to the control group with no significant difference (*p* > .05), while Simpson and Shannon values were significantly decreased (*p* < .01, *p* < .05). Compared with the model group, the Chao1, Simpson, and Shannon values were increased in the RRP group with no significant difference (*p* > .05). PCOA and NMDS analysis of β-diversity showed that the colony structure was independently separated between the control and model groups, while there was an overlap between the control and RRP groups (Figures 5B, C) indicating that the microbiota composition was more inclined to that of the control group.

#### 3.5.2 Species distribution of intestinal microbiota in each group

Figure 5D displayed Genus-level species composition heatmap for species clustering. Figure 5E–H showed the significant differences in the abundance of ASVs/OTU sequences at phylum and class levels among different groups. The results showed that the abundance of Bacteroidetes, Clostridia and Bacteroidia were significantly decreased (*p* < .001, *p* < .001, *p* < .001) and Firmicutes and Bacill were significantly increased (*p* < .01, *p* < .001) in model group compared to control group. After RRP treatment, the levels of Bacteroidetes, Bacteroidia were significantly increased significantly (*p* < .001, *p* < .001), while the levels of Firmicutes and Bacill were significantly decreased significantly in mice of RRP group compared to model group (*p* < .001, *p* < .001).

### 3.6 Analysis of compounds in RRP extracted powder

**FIGURE 6:**
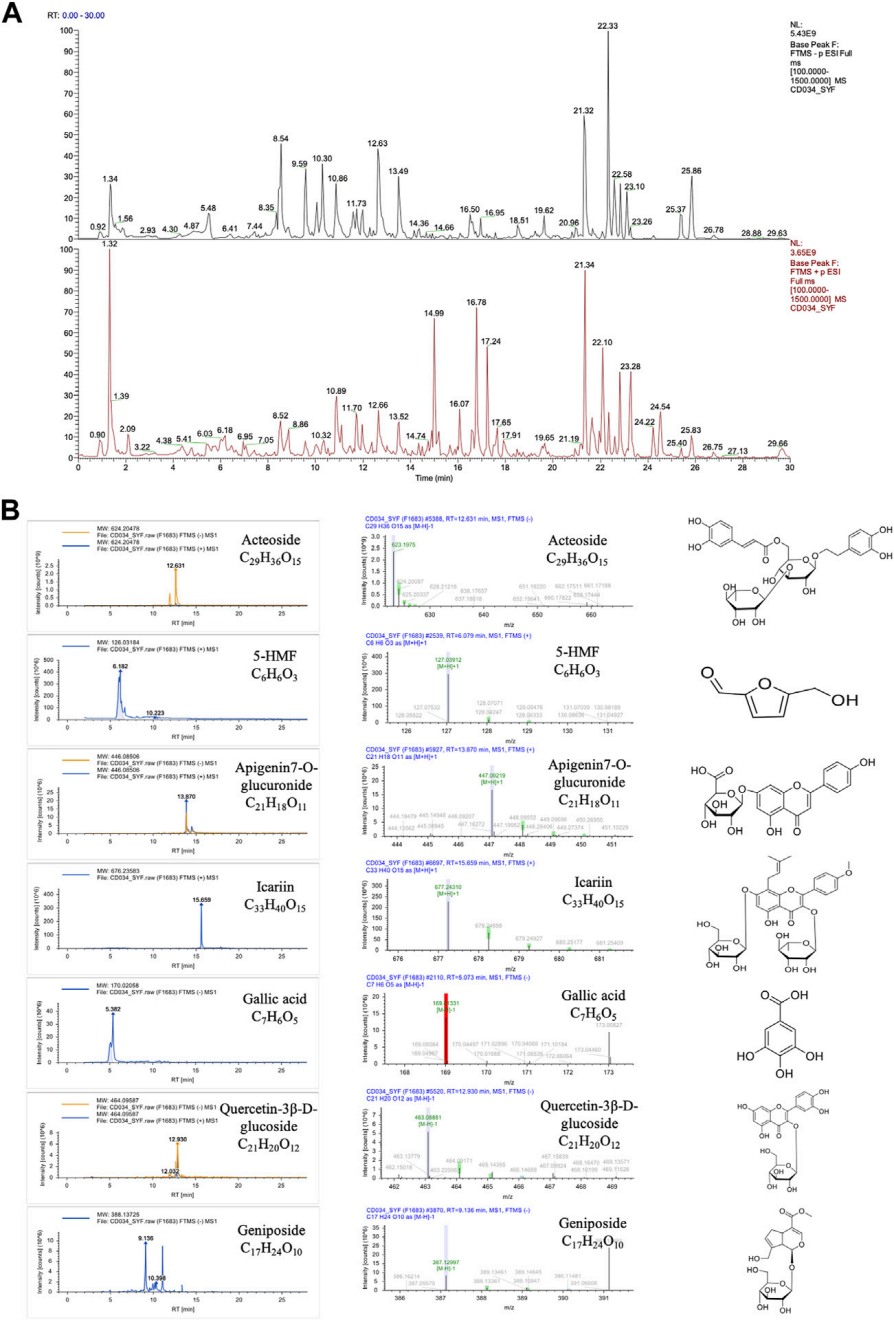
Compounds in the RRP. **(A)**. Total ion chromatogram of compound identification; **(B)**. Mass chromatograms (in positive and negative mode) and structure of Acteoside, 5-HMF, Apigenin7-O-glucuronide, Icariin, Gallic acid, Quercetin-3β-D-glucoside and Geniposide.

The structure of compounds was identified by mass spectrometry. Ten compounds in the sample had a combined score of >80 in the mzCloud best match, and seven of which were reported in the RRP. These seven compounds were Acteoside (Verbascoside), 5-Hydroxymethyl-2-furaldehyde (5-HMF), Apigenin7-O-glucuronide, Icariin, Gallic acid, Quercetin-3β-D-glucoside, and Geniposide respectively. The chemical formula and structure are shown in Figure 6.

### 3.7 Analysis of the binding ability of the compounds in RRP to INSR protein

**FIGURE 7:**
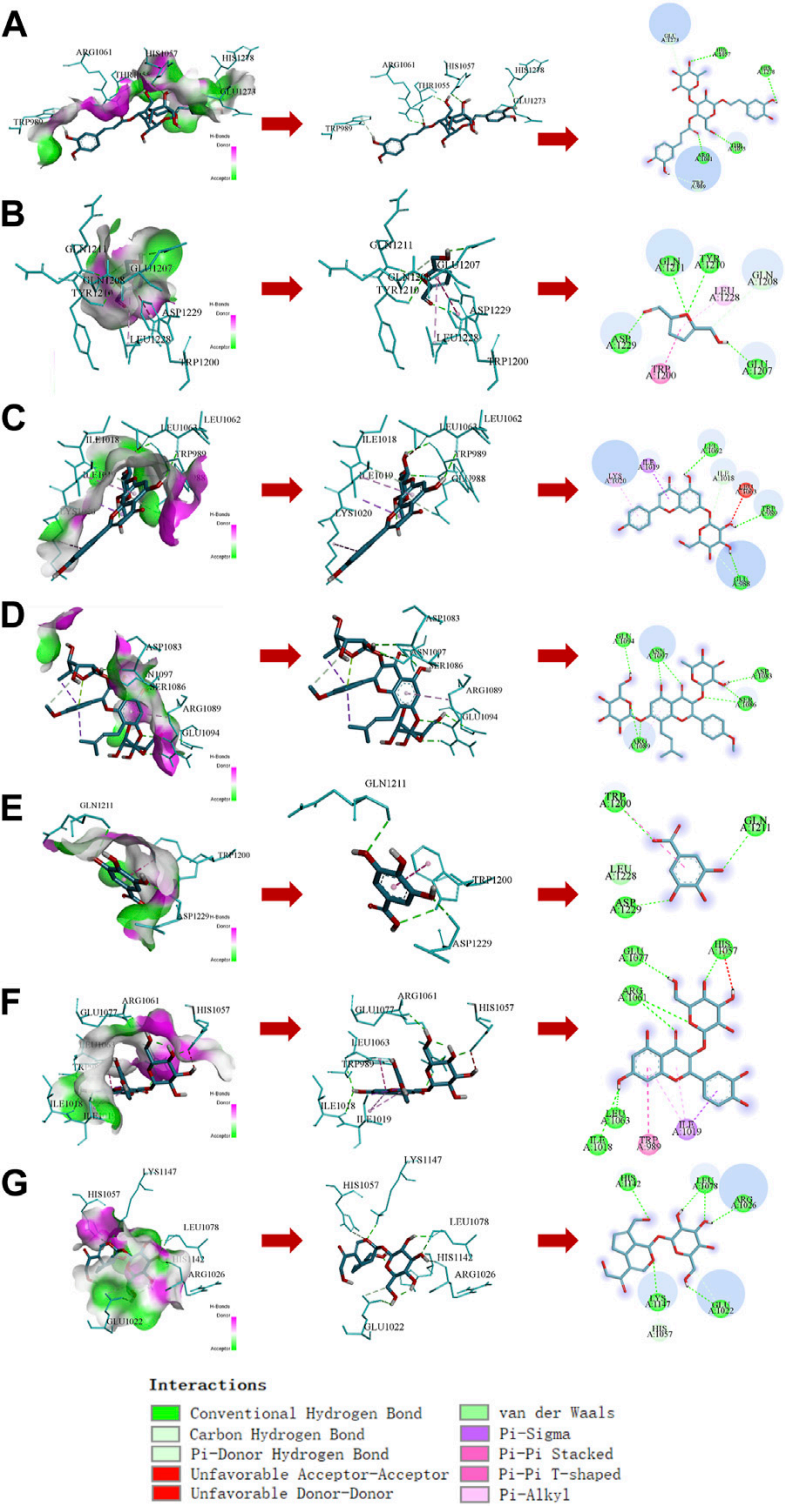
Docking analysis of compounds in RRP with INSR protein. **(A–G)**: Acteoside, 5-HMF, Apigenin7-O-glucuronide, Icariin, Gallic acid, Quercetin-3β-D-glucoside, Geniposide.

The molecular docking results showed that compounds in RRP could bind to INSR protein, among which the binding energy of Icariin to INSR protein was −9.7 kcal mol^−1^ eight hydrogen bonds were formed with GLU1094, ASN1097, ARG1089, ASP1083, and WER1086. Different compounds bind to INSR proteins at different sites. The number of hydrogen bonds formed by the compounds in RRP with INSR proteins, the minimum binding energy and the binding sites are shown in Figure 7 and Table 1.

**TABLE 1:** Summary of compounds binding to INSR protein in the RRP.

No.	Compound name	Tcmsp no.	Total number of formed hydrogen bonds	Lowest binding energy (kcal·mol^-1^)	Binding sites
1	Acteoside	MOL003333	5	−9.3	HIS1057, HIS1278, THR1055, ARG1061
2	5-HMF	MOL000748	4	−4.4	ASP1229, GLN1211, TYR1210, GLU1207
3	Apigenin7-O-glucuronide	MOL000007	3	−8.0	LEU1062, TRP989, GLU988
4	Icariin	MOL004425	8	−9.7	GLU1094, ASN1097, ARG1089, ASP1083, WER1086
5	Gallic acid	MOL000513	3	−5.6	TRP1200, ASP1229, GLN1211
6	Quercetin-3β-D-glucoside	MOL000437	6	−8.7	HIS1057, GLU1077, ARG1061, LEU1063, ILE1018
7	Geniposide	MOL003701	5	−8.0	HIS1142, LEU1078, ARG1026, GLU1022, LYS1147

## 4 Discussion

The pathogenesis of AD is complex and clinical medications are lacking (Cummings et al., 2020). Traditional Chinese medicine has a long history of understanding AD and has natural advantages in improving cognitive impairment and delaying the development of AD. It has been found that brain insulin signaling impairment begins early in AD and accompanies the course of the disease and worsens as the disease progresses (Rivera et al., 2005). As a major pathological feature of T2DM, abnormal peripheral insulin signaling is widely recognized as an important risk factor for the development of AD (Kim and Feldman, 2015) and is involved in the development of non-type 2 diabetic AD patients (Femminella et al., 2021).

RRP is widely used as primal medicine in Chinese herbal formula for the treatment of AD or diabetes, for example, Qi Fu Yin, Qidan Dihuang decoction, Zhibai Dihuang Wan, Dihuang Yinzi, and Liuwei Dihuang (Chen et al., 2018; Ong et al., 2018; Lu et al., 2020; Lee et al., 2021; Song et al., 2022). Several studies have reported that the components in RRP are effective against AD (Huang et al., 2016; Kang et al., 2022). In this study, we investigated the protective effects and potential mechanisms of RRP in ICV-STZ-induced AD mice to provide a basis for further clinical application of RRP.

In the water maze experiment, the escape latency is the time required for mice to successfully find the platform for the first time after each entry into the water, and a short latency period predicts good learning memory ability of mice. The longer the time elapsed in the target quadrant and the greater the number of crossings, the better the spatial learning and memory ability of the animal. The open field experiment can be used to observe the autonomous locomotor ability of experimental animals. The total distance moved and the number of grooming are the stress behaviors produced in response to the new and different environment, indicating that the mice are more excited and have better autonomous locomotor ability at this time. The alternation triplet in the Y-maze experiment indicated the spatial exploration ability of mice, and the high alternation rate meant that the mice had better spatial exploration ability. The results of behavioral tests showed that RRP treatment decreased the escape latency and increased the elapsed time in targeted quadrant and the number of crossings in water maze experiment, increased the total distance and grooming number in open field test and alternation triplet in Y-maze experiment in AD mice, indicating that RRP improved the cognitive dysfunction of AD mice. H&E staining showed that pathological damage in the brain was reduced in the RRP-treated group of mice, suggesting a neuroprotective effect of RRP in AD mice. AD is the most common neurodegenerative disease and hyperphosphorylation of tau protein is considered as one of the pathological markers (Drummond et al., 2020; Ossenkoppele et al., 2022). Ser404 is a common tau hyperphosphorylation site (Yang et al., 2020). Western-blot and IF staining results showed that RRP decreased ICV-STZ-induced accumulation of p^Ser404^-tau protein in the brain tissue of AD mice, indicating that RRP improved the pathology of AD.

Insulin signaling is considered to be a common link connecting many other AD hypotheses, which is a hot spot of research for anti-AD drug development (Alves et al., 2021; Tyagi and Pugazhenthi, 2021). In the present study, we found that RRP increased the protein expression levels of INSR, IRS-1, p^Ser473^-AKT/AKT, and p^Ser9^-GSK-3β/GSK-3β in brain tissue, indicating that RRP restored the insulin signaling pathway in the brain of AD mice. It is not clear which components of RRP can regulate the insulin signaling pathway. Seven compounds were identified from the extracted powder of RRP by mass spectrometry analysis. According to the reports, Acteoside (Chen et al., 2021), 5-HMF (Liu et al., 2014), Icariin (Khezri and Ghasemnejad-Berenji, 2022), Gallic acid (Mori et al., 2020), and Geniposide (Zhang et al., 2021) are potential neuroprotective drugs for AD. Docking analysis showed that the compounds have some binding ability to INSR protein with different binding sites, indicating potential multiple synergistic effects. However, which components of RRP play regulatory roles needs to be further verified by experiments.

Intestinal microbiota imbalance is closely associated with AD pathogenesis (Pluta et al., 2020). Recent studies have found that targeting the intestinal microbiota holds good promise against AD (Sun et al., 2019; Wang et al., 2019; Kim et al., 2020; van Olst et al., 2021). Therefore, we investigated the effect of RRP on the intestinal microbiota of AD mice. Alpha diversity showed significantly lower Simpson and Shannon values in the model group, indicating reduced microbiota abundance and diversity in AD mice. Interestingly, beta diversity showed that RRP restored the microbiota structure in AD mice. Firmicutes and Bacteroidetes are the largest components of intestinal microbiota. After RRP treatment, the intestinal microbiota structure of AD mice tended to healthy levels, with reversal of Bacteroidetes, Bacteroidia, Firmicutes, and Bacill levels. Intestinal microbiota contributes to peripheral and central insulin homeostasis and action (Schertzer and Lam, 2021) as well as neurobehavioral regulation (Soto et al., 2018). Intestinal microbiota imbalance is common in AD patients (van Olst et al., 2021) and has been reported in APP/PS1 transgenic mice and 5✕FAD transgenic mice (Zhang et al., 2017; Liu et al., 2021). Studies have shown that restoring the intestinal microbiota is effective in improving AD (de Rijke et al., 2022; Snigdha et al., 2022). Therefore, the mechanism of improving AD by modulating insulin signaling in the brain may be related to restoring the intestinal microbiota. Further reverse validation of RRP in AD gut microbiota is needed, such as gut flora transplantation.

## 5 Conclusion

In summary, it is concluded that RRP significantly improved cognitive dysfunction and brain pathological changes in AD mice. The mechanism of RRP improvement in AD may be related to the regulation of INSR/IRS-1/AKT/GSK-3β signaling pathway and intestinal microbiota. This study provides a theoretical basis for the clinical application of RRP. However, this study is limited in that only one animal model of AD was selected for study and no reverse validation was performed. In the future, transgenic mouse models of AD and reverse validation at the cellular level is need to be tested. In addition, the further identification of active ingredients in RRP will provide more scientific basis for the development of new anti-AD drugs.

## Data Availability

The original contributions presented in the study are all included in the article/Supplementary Material, further inquiries can be directed to the corresponding author.

## Abbreviations

RRP, Radix Rehmanniae Praeparata; INSR, insulin receptor; ICV-STZ, intraventricular injection of streptozotocin.
